# Impact of Grinding Depth on Dislocation Structures and Surface Hardening in C45 Steel

**DOI:** 10.3390/ma18163870

**Published:** 2025-08-18

**Authors:** Alicja Stanisławska, Dorota Moszczyńska, Jarosław Mizera, Pasquale Cavaliere, Marek Szkodo

**Affiliations:** 1 Faculty of Mechanical Engineering and Ship Technology, Gdansk University of Technology, Narutowicza 11/12, 80-233 Gdańsk, Poland; 2 Faculty of Materials Science and Engineering, Warsaw University of Technology, Wołoska 141, 02-507 Warsaw, Poland; dorota.moszczynska@pw.edu.pl (D.M.); jaroslaw.mizera@pw.edu.pl (J.M.); 3 Department of Innovation Engineering, University of Salento, Via per Arnesano, 73100 Lecce, Italy; pasquale.cavaliere@unisalento.it

**Keywords:** C45 steel, grinding, residual stress, dislocation structure, dislocation mobility

## Abstract

This study investigates the strain hardening and dislocation structure in the surface layers of C45 steel subjected to precision grinding at various depths. The aim was to assess how different grinding conditions influence the mechanical response and defect structure of ferrite. Nanoindentation was used to evaluate mechanical properties, while X-ray diffraction analysis provided data on the microstrain, crystallite size, and residual stress. The character and density of dislocations were further examined using modified Williamson–Hall and q-parameter analysis. The results revealed that the sample ground to a depth of 2 μm exhibited the highest density of statistically stored dislocations, as well as the lowest dislocation mobility. This condition also corresponded to the highest residual stresses and the greatest share of screw dislocations, indicating intense strain localization. In contrast, deeper grinding depths resulted in lower dislocation densities and reduced the strain energy. The observed trends highlight the formation of a dislocation-rich nanostructured layer in the shallowest ground region. These findings provide new insights into the mechanisms of surface hardening in ferritic steels and demonstrate how the depth of material removal during grinding governs the subsurface microstructure and strengthening effects.

## 1. Introduction

Grinding is widely used not only for dimensional shaping and surface finishing but also as a means to modify the subsurface microstructure of metallic materials. The interaction between abrasive grains and the workpiece generates both mechanical and thermal effects, including plastic deformation, frictional heating, and material removal through micro-cutting and ploughing. When the depth of the cut is very small, the energy dissipated via plastic deformation and friction can exceed the energy required for chip formation, providing favorable conditions for strain hardening of the surface layer [1,2,3].

However, for this hardening to be effective and not reversed by thermal effects, the surface temperature during grinding must be carefully controlled. At low grinding speeds and shallow depths, temperatures typically remain below 300 °C [4], which is significantly lower than the austenitization threshold of approximately 728 °C for C45 steel. This avoids undesirable phase transformations while promoting high-density dislocation structures in the near-surface region. Such a mechanism allows grinding to act as a sustainable alternative to traditional thermochemical hardening techniques such as quenching, carburizing, or nitrocarburizing.

These traditional methods, although effective in increasing surface hardness, often involve substantial energy input, long processing times, and environmental risks. For instance, quenching C45 steel typically requires heating to 820–860 °C followed by rapid cooling in oil or water, resulting in surface hardness up to 58 HRC (around 650 HV) and the formation of a martensitic layer. Carburizing processes demand several hours at 900 °C to achieve surface hardness in the range of 700–800 HV, while nitrocarburizing also uses elevated temperatures and reactive atmospheres to induce hardness values around 600–750 HV in the diffusion layer [5]. These methods are not only resource-intensive but also rely on toxic media, such as quenching oils or ammonia-based gases, that pose occupational and ecological hazards.

Grinding, particularly when conducted with carefully selected parameters, enables surface strengthening through purely mechanical means, eliminating the need for high temperatures or chemical treatments. Moreover, it avoids the disposal of harmful quenching oils and reduces operator exposure to toxic substances [5]. As such, it aligns with the principles of green manufacturing. Despite these advantages, the microstructural mechanisms governing strain hardening during shallow grinding remain underexplored. In our earlier work [6], we demonstrated a correlation between the grinding depth and surface hardness in C45 steel. However, the influence of grinding on dislocation structures, residual stress development, and defect mobility is still poorly understood.

In this study, we hypothesize that the depth of grinding directly affects the formation and mobility of dislocations in the surface layer of C45 steel, with shallower depths promoting higher dislocation densities, increased screw dislocation fractions, and more pronounced residual stresses, leading to enhanced strain hardening. The objective is to characterize these microstructural changes and correlate them with the mechanical response of the material.

To address this, we performed nanoindentation tests and X-ray diffraction (XRD) analyses, including modified Williamson–Hall and q-parameter evaluations, to assess the dislocation density, character, and residual stress. Electron backscatter diffraction (EBSD) was used to examine crystallographic misorientation near the surface. These complementary methods provide a comprehensive understanding of how precision grinding parameters influence the dislocation-mediated mechanisms responsible for strain hardening.

The novelty of this research lies in its integrated, quantitative assessment of the relationship between the grinding depth and dislocation-based surface strengthening in ferritic steel. Unlike previous studies that focused mainly on surface roughness or hardness values, this work provides mechanistic insight into the evolution of dislocation structures under shallow grinding conditions. The results may contribute to the development of environmentally friendly, oil-free alternatives to conventional hardening techniques, with potential applications in precision machining and surface engineering.

## 2. Materials and Methods

### 2.1. Workpiece Preparation

The test specimens were cylindrical, with a diameter of 50 mm and a height of 10 mm, and were cut from a rod of the same diameter using wire electrical discharge machining (EDM). Prior to cutting, a 150 mm long section of the rod was normalized in a vacuum furnace at 850 °C for 20 min, followed by air cooling to ambient temperature. Metallographic examination after heat treatment confirmed a microstructure composed of ferrite and pearlite, with an average grain size of approximately 20 μm.

The chemical composition of the steel was determined as the average of three measurements using an OE750 spark optical emission spectrometer. The chemical composition of the C45 steel used in this study is presented in Table 1.

After normalization, the steel exhibited a Brinell hardness of 167 HB and a tensile strength of 490 MPa.

### 2.2. Grinding the Workpiece

The grinding process was carried out using a CNC surface grinder with a horizontal spindle axis (SPG 25 × 60) equipped with a Norton grinding wheel (38A60LVS) of dimensions 250 × 25 × 76.2 mm. Four single grinding passes were performed on each specimen (two passes per side). The grinding wheel operated at a constant circumferential speed of v_s_ = 25 m/s, while the table feed rate was fixed at v_ft_ = 1 m/min. Each pass was carried out at a different grinding depth: a_e_ = 2 μm, 8 μm, 14 μm, and 20 μm, respectively. A summary of all grinding parameters is presented in Table 2. The grinding parameters were selected based on the literature data and preliminary tests to avoid thermal damage and ensure reproducible results [6,7].

During grinding, a water-based coolant was applied at a flow rate of 4.3 L/min. The coolant consisted of water and Blasocut 2000 Universal mineral oil mixed in a volume ratio of 1:20.

Before each grinding pass, the wheel was conditioned using a single-point diamond dresser. The dressing parameters were as follows: number of spark-out passes: 2; number of cross-feed passes: 4; lateral feed rate: 0.2 mm/rev; peripheral speed of the wheel during dressing: 23 m/s; and depth of cut per pass: 0.1 mm.

### 2.3. Characterization

To evaluate the residual stresses and crystallite size within the ferrite grains of the ground surface layer, X-ray diffraction (XRD) analysis (X’Pert diffractometer, using CuKα radiation) was conducted using the Williamson-Hall (W-H) method. A modified W-H approach was further applied to the XRD patterns to determine the relative fractions of the screw and edge dislocations in the ferrite matrix.

Dislocation density was assessed using the nanoindentation technique, which offers the advantage of probing a larger material volume compared to transmission electron microscopy (TEM), which provides localized information. Additionally, the nanoindentation tests enabled the assessment of dislocation mobility.

The microstructure of the ground surface layer was examined using a JEOL 7800F scanning electron microscope (SEM, JEOL JSM-7800F, JEOL Ltd., Tokyo, Japan). Detailed crystallographic characterization was carried out by electron backscatter diffraction (EBSD, camera was manufactured by Oxford Instruments, High Wycombe, United Kingdom). EBSD scans were performed at an accelerating voltage of 20 kV with a step size of 0.2 μm. Microstructural maps were generated using HKL Channel 5 software (version 5.12, Oxford Instruments, Abingdon, UK).

#### 2.3.1. XRD Analysis for Lattice Strain and Crystallite Size

X-ray diffraction (XRD, PANalytical B.V., Almelo, The Netherlands) measurements of the ground surface layer were performed using a Cu anode tube, emitting radiation with a wavelength of 0.15418 nm. The diffractometer operated at an accelerating voltage of 30 kV and a tube current of 50 mA. Scans were conducted over a 2θ range from 20° to 90°, with a step size of 0.02° and a counting time of 5 s per step. The diffraction peaks were fitted using Lorentzian functions via nonlinear least-squares regression in Origin software.

To determine the crystallite size and lattice microstrain in ferrite grains of ground C45 steel, the Williamson–Hall (W-H) method was applied [8]. This method assumes that peak broadening in the diffraction pattern arises from two main factors: (1) the finite size of coherently diffracting domains (crystallites) and (2) lattice distortions (microstrains). Additionally, instrumental broadening intrinsic to the measurement system must be accounted for.

The total broadening B (in radians) can thus be expressed as follows:

B = B_L_ + B_s_ + B_i_ (1)

where B denotes the total observed peak broadening, composed of three contributions: broadening due to finite crystallite size B_L_, microstrain-induced broadening B_s_, and instrumental broadening B_i_. The size-related broadening can be described using the Scherrer equation B_L_ = a_s_⋅λ/L⋅cosθ where a_s_ is a shape factor dependent on the crystallite geometry, λ is the X-ray wavelength, L is the effective crystallite size, and θ is the Bragg angle. The contribution from lattice microstrains is expressed as B_s_ = 4ε ⋅tanθ where ε represents the root-mean-square lattice strain. Instrumental broadening, determined using a high-purity silicon reference sample, was found to be approximately 8% of the measured FWHM for the diffractometer configuration used in this study. Taking all contributions into account, Equation (1) can thus be reformulated as follows:

B⋅cosθ = a_s_⋅λ/L + 4ε⋅sinθ (2)

The peak position and broadening uncertainties were determined from Lorentzian fitting residuals and propagated to the calculation of the microstrain, crystallite size, and residual stress. The relative uncertainty of these parameters was estimated at ±5% for the microstrain and ±10% for the crystallite size.

#### 2.3.2. XRD Analysis for Dislocation Structure

To evaluate the dislocation structure in the strain-hardened surface layers, a modified Williamson–Hall (MW-H) analysis was employed. In this method, diffraction peak broadening is analyzed as a function of the diffraction vector K = 2sinθ/λ where θ is the Bragg angle and λ is the X-ray wavelength. As in standard WH analysis, peak broadening was determined using Lorentzian peak fitting in Origin software (version 9.0, OriginLab Corporation, Northampton, MA, USA), preceded by peak deconvolution to improve accuracy.

Ferrite, with its body-centered cubic (BCC) crystal structure, exhibits significant elastic anisotropy. For such materials, the MW-H equation is expressed as follows [9]:

(3) $$\Delta K≅\frac{a_{s}}{L}+bM\sqrt{\frac{\pi }{2}\rho \left(K{\bar{C}}^{1/2}\right)}$$

where ΔK represents the full width at half maximum (FWHM) of the diffraction peaks plotted against the diffraction vector K. The parameter M is a dimensionless coefficient that reflects the spatial arrangement of dislocations within the material. The symbols ρ and b correspond to the dislocation density and the magnitude of the Burgers vector, respectively. The term C denotes the average dislocation contrast factor, which can be determined using the following expression [9].

(4) $$\bar{C}={\bar{C}}_{h00}\left(1-qH^{2}\right)$$

Here, C_h00_ is the average contrast factor for the {h00} reflections, which depends on the type of dislocation (edge or screw), and q is a parameter reflecting the proportion of screw and edge dislocations. The geometric factor H^2^ is defined based on Miller indices of the reflecting planes:

(5) $$H^{2}=\frac{h^{2}l^{2+}h^{2}k^{2}+l^{2}k^{2}}{{\left(h^{2}+k^{2}+l^{2}\right)}^{2}}$$

By combining Equations (3) and (4) and rearranging them, a linear relationship is obtained [10]:

(6) $$\frac{{\left(∆K-\alpha \right)}^{2}}{K^{2}}≅\beta^{2}{\bar{C}}_{h00}\left(1-qH^{2}\right)$$

where α = a_s_/L and β = *bM*(*πρ*/2)^1/2^.

Plotting (ΔK − α)^2^/K^2^ against H^2^ yields a straight line. The H^2^ intercept of this line corresponds to 1/q, from which the experimental value of the dislocation character parameter q is obtained.

For BCC structures, theoretical values of q are 1.298 for pure edge dislocations and 2.686 for pure screw dislocations. The relative fractions of edge and screw dislocations can be estimated using the lever rule [11] as follows:

(7) $$f^{edge}=\frac{q_{screw}^{th}-q}{q_{screw}^{th}-q_{edge}^{th}}=1-f^{screw}$$

where q is the experimentally determined dislocation character parameter, and f_edge_ and f_screw_ denote the fractions of edge and screw dislocations, respectively.

#### 2.3.3. Nanoindentation Test

Prior to hardness testing, a 5 mm wide section was cut from the central part of the workpiece along its diameter, perpendicular to the grinding direction. This section was halved and each part embedded in thermosetting resin. Wire electrical discharge machining was used to cut the specimens, resulting in two cross-sections with oppositely ground surfaces at varying depths. Each cross-section was manually ground using SiC abrasive papers ranging from 60 to 2000 grit, followed by mechanical polishing with diamond paste. To eliminate the Beilby layer, the surfaces were repeatedly etched with a 3.5% nitric acid solution in ethanol and polished under progressively lower loads.

Nanoindentation tests were conducted using a Berkovich diamond indenter under a maximum load of 20 mN, applied at a rate of 1 mN/s, with a 5 s holding time at peak load followed by unloading over 15 s. The Berkovich tip has a three-sided pyramidal geometry equivalent to a right circular cone with a semi-apex angle of 65.27° in terms of the projected area-to-depth ratio. The projected contact area A was calculated using the Oliver–Pharr method as A = 24.5 h^2^_c_, where h_c_ is the contact depth determined from the load–displacement curve. Measurements were taken within ferrite grains located in the strain-hardened layer, specifically within 5 μm of the ground surface, avoiding grain boundaries and defects. For each grinding depth, three independent measurements were performed in ferrite grains, and the mean hardness and modulus values were calculated along with standard deviations. Statistical significance of differences between depths was assessed using one-way ANOVA (*p* < 0.05). All tests were performed at a constant ambient temperature of 21 ± 0.5 °C to minimize thermal effects.

To assess the hardness and stiffness gradient of the surface layer, a depth-sensing indentation procedure was applied directly to the ground surface. A series of 10 indentations with increasing loads ranging from 100 to 2000 mN were performed at different depths (2–10 μm from the surface). In each cycle, the load was applied over 20 s, held for 5 s, and unloaded over the following 20 s.

**Figure 1:**
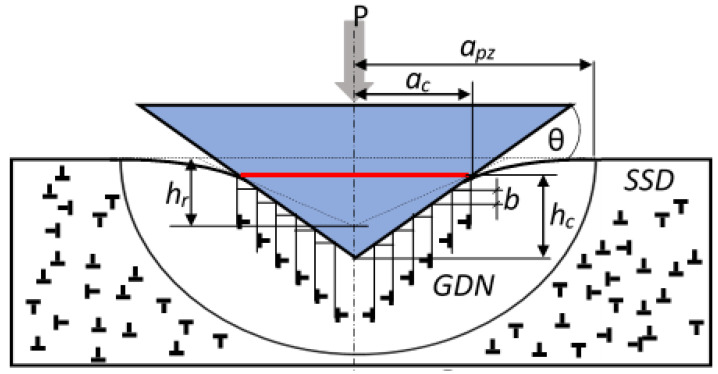
The geometry of a conical indenter contacting an elastic–plastic material. A semispherical plasticized zone with a selected GND has been marked. The red line indicates the contact surface.

During indentation, geometrically necessary dislocations (GNDs) form to accommodate plastic deformation beneath the indenter and allow for lattice curvature. These GNDs accumulate within the plastically deformed zone and interact with statistically stored dislocations (SSDs) generated during grinding. The density of GNDs is estimated using the following relation [12]:

(8) $$\rho_{GND}=\frac{3}{2}\cdot \frac{1}{f^{3}}\cdot \frac{{tan}^{2}\theta }{bh_{c}}$$

where θ = 19.7° is the equivalent cone angle for the Berkovich indenter, b = 0.284 nm is the Burgers vector for ferrite, h_c_ is the contact depth, and f = a_pz_/a_c_ is the ratio of the plastic zone radius to the contact radius (Figure 1). For ferrite, the plastic zone radius is typically more than twice the contact radius, and f = 2.2 was used based on [13].

Although Equation (8) assumes conical geometry, it remains valid for Berkovich indenters due to the equivalence in the projected area-to-depth ratio. A smaller contact depth corresponds to a higher GND density, which, according to Taylor’s relation, results in increased hardness, a phenomenon known as the indentation size effect (ISE). In addition to GNDs, the ISE can also be influenced by material anisotropy [14], internal friction (H_fr_), grain boundary hardening (H_gb_), and solid solution strengthening (H_ss_) [15], though these effects were neglected in the present study.

The overall hardness incorporating the ISE can be expressed as follows:

(9) $$H_{ISE}=H_{fr}+{H_{gb}+H}_{ss}+MC\alpha Gb\sqrt{\rho_{GND}+\rho_{SSD}}$$

where M = 3 is the Taylor factor, C = 3 is the constraint factor [16], and α = 0.5 accounts for the dislocation structure. For ferrite, G = 80 GPa.

In contrast, the macroscale hardness (i.e., without the ISE) is described by a simplified Taylor relationship [17]:

(10) $$H_{0}\;=\;\mathrm{MC}\alpha \mathrm{Gb}\cdot \sqrt{\rho_{\mathrm{SSD}}}$$

This distinction allows the quantification of both strain-induced and inherent dislocation contributions to the surface hardening observed after grinding.

#### 2.3.4. Nanoindentation Test for Dislocation Mobility Measurement

The average dislocation velocity was estimated based on nanoindentation data by applying the Orowan equation [18,19]:

(11) $$\dot{\varepsilon }=b\cdot \rho_{SSD}\cdot \upsilon$$

where $\dot{\varepsilon }$ is the plastic strain rate, b is the Burgers vector, ρ_SSD_ is the density of statistically stored dislocations, and υ is the average dislocation velocity.

The strain rate $\dot{\varepsilon }$ was determined from the displacement–time (h-t) curve recorded during the holding (dwell) period of the nanoindentation test under constant load. This curve typically exhibits two distinct regions. In the initial phase, the strain rate decreases due to stress relaxation and material accommodation. In the subsequent phase, the curve reaches a quasi-steady state, during which the strain rate stabilizes. This second, linear segment was used to calculate $\dot{\varepsilon }$, ensuring consistency and minimizing transient effects.

By substituting the measured strain rate and dislocation density into Equation (11), the average dislocation velocity υ in the strain-hardened surface layer was obtained. This parameter provides insight into the mobility of dislocations under stress and complements the characterization of the dislocation structure derived from the modified Williamson–Hall and hardness analyses.

## 3. Results

**Figure 2:**
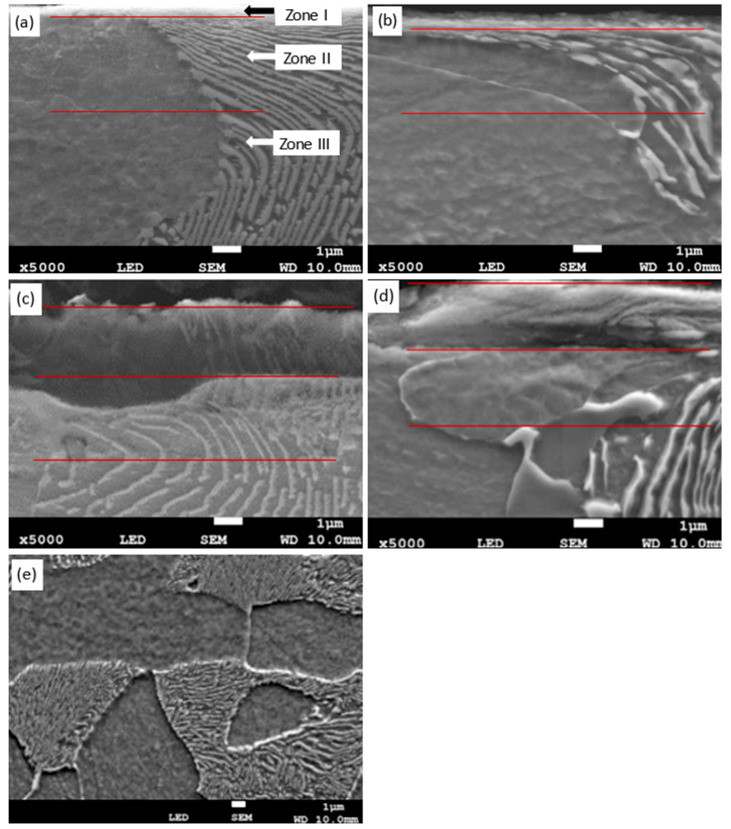
Microstructure of the surface layer of C45 steel after grinding to depths of 2 µm (**a**), 8 µm (**b**), 14 µm (**c**), and 20 µm (**d**), showing the evolution of three characteristic sublayers. For comparison, the microstructure of untreated ferrite–pearlite steel is shown in (**e**). Three distinct sublayers are identified in the ground samples. Zone I: the outermost layer, thermally affected and partially oxidized, showing a dense and compact morphology due to severe surface interactions during grinding. Zone II: a plastically deformed subsurface region with refined ferritic grains, high internal misorientation, and dense dislocation structures. Zone III: a transition zone resembling the bulk microstructure, composed of equiaxed ferrite with low deformation features. The boundary between Zones II and III was determined based on EBSD analysis, particularly a sharp decrease in grain orientation spread (GOS) and the disappearance of deformation substructures.

**Figure 3:**
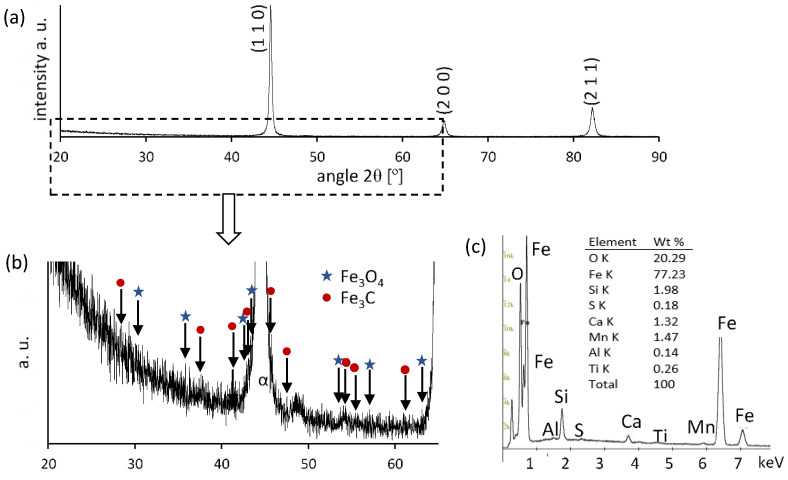
(**a**,**b**) XRD pattern and (**c**) EDS image for the surface layer ground to a depth of 2 µm.

**Figure 4:**
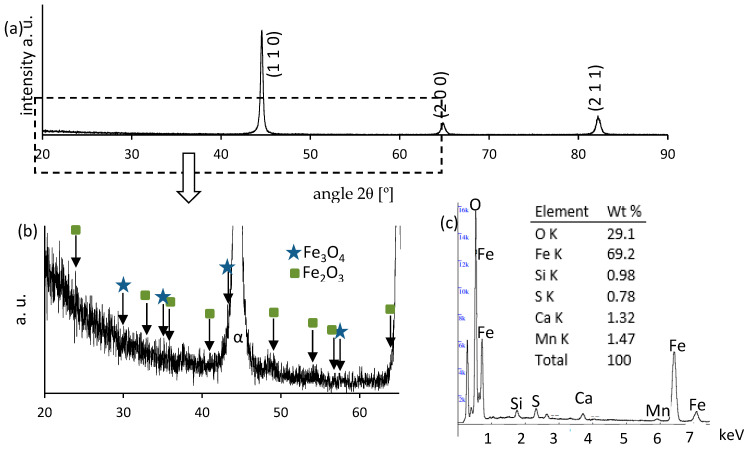
(**a**,**b**) XRD pattern and (**c**) EDS image for the surface layer ground to a depth of 20 µm.

Figure 2 presents the microstructure of the surface layer of C45 steel after grinding to depths ranging from 2 to 20 µm. In each case, three distinct sublayers are visible:

- Zone I—the outermost layer, enriched in oxides, as confirmed by XRD and EDS analyses (Figure 3 and Figure 4);
- Zone II—plastically deformed ferrite and pearlite grains;
- Zone III—the unaffected base microstructure.

The interlamellar spacing in pearlite is significantly reduced in Zone II compared to in Zone III, indicating intensive plastic deformation. Increasing the grinding depth results in a thicker oxide layer (Zone I) and a thinner deformation zone (Zone II). At a 2 µm depth, the oxide consists mainly of magnetite (Fe_3_O_4_), while at 20 µm, a mixture of magnetite and hematite (Fe_2_O_3_) appears. The presence of hematite suggests a higher surface temperature, but the absence of wüstite (FeO) implies that it did not exceed 570 °C. No refinement of pearlite grains was observed, indicating that the pearlite-to-austenite transformation threshold of ~728 °C was not reached.

**Figure 5:**
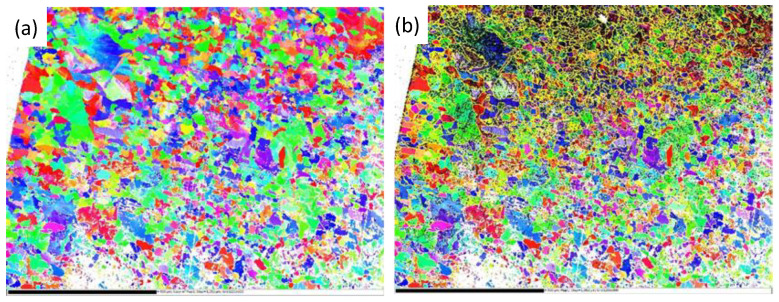
(**a**) Crystallographic orientation of grains in the surface layer of C45 steel after grinding to a depth of 2 µm and (**b**) grain and subgrain boundaries in the surface layer.

The most compact and adherent oxide layer formed at a 2 µm depth, which is considered the most beneficial in terms of microstructural integrity. EBSD analysis (Figure 5) revealed a pronounced crystallographic texture and ferrite grain refinement, consistent with plastic deformation. Both low-angle and high-angle boundaries were observed, indicating heterogeneous deformation mechanisms. Elevated dislocation densities further confirm intense strain hardening and suggest the presence of residual stresses.

**Figure 6:**
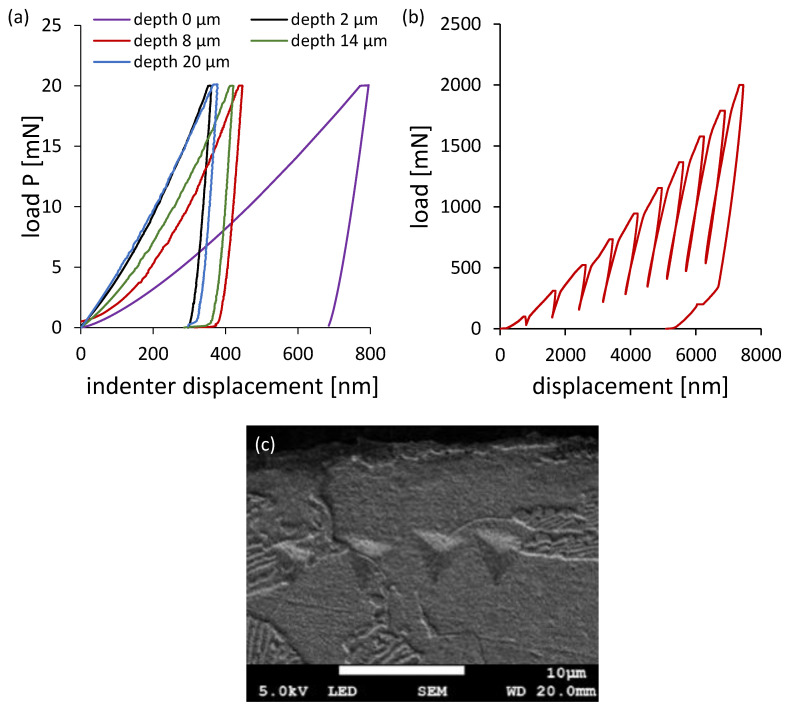
(**a**) Load–displacement curves obtained from the test performed on the cross-section of ground surface layers and (**b**) the curve obtained in multiload mode on the surface of steel ground to a depth of 2 μm. (**c**) This shows indents in a ferrite grain after the nanoindentation test on the cross-section of steel ground to a depth of 20 μm.

To investigate the mechanical response, nanoindentation tests were performed (Figure 6). The tests revealed increased hardness and contact stiffness within approximately 5 µm of the surface, particularly for grinding depths of 2 µm and 20 µm. Using the Oliver–Pharr method and Sneddon’s solution, the indentation hardness (H_ISE_) and reduced Young’s modulus (E*) were determined. These values, along with the geometrically necessary dislocation density (ρ_GDN_), statistically stored dislocation density (ρ_SSD_), and macroscopic hardness (H_0_), are summarized in Table 3. The data confirm that the highest dislocation densities occurred at 2 µm and 20 µm, with values of 4.716 × 10^15^ m^−2^ and 3.740 × 10^15^ m^−2^, respectively.

**Figure 7:**
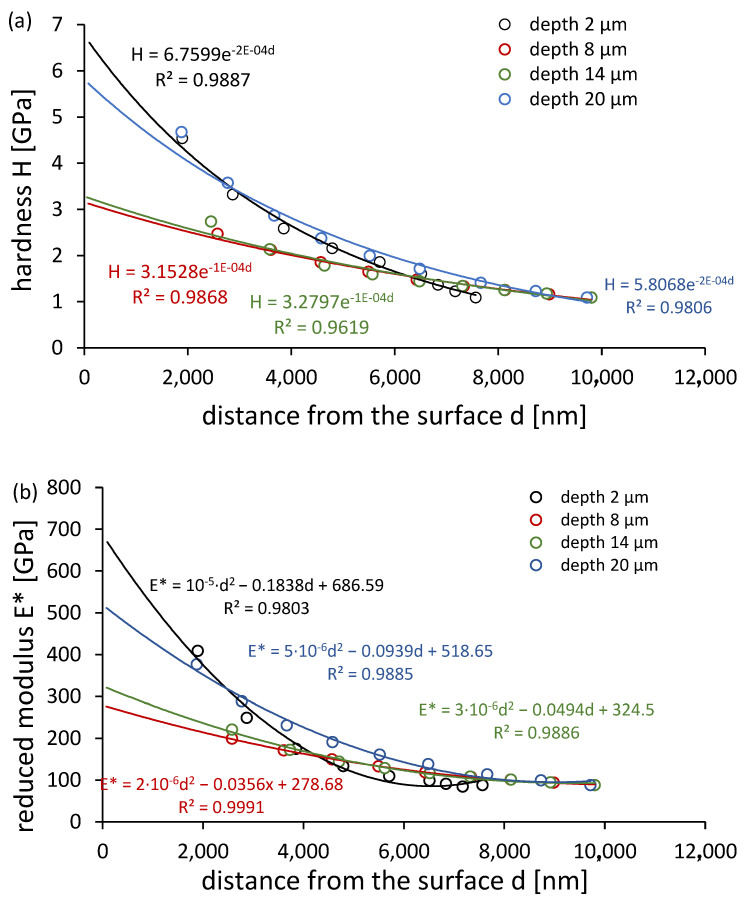
(**a**) Hardness distribution profiles and (**b**) stiffness distribution profiles for surface layers after grinding to different depths, determined in the nanoindentation test in multiload mode.

Hardness and stiffness distribution profiles were further assessed using multiload nanoindentation mode (Figure 7). The hardness depth profiles followed an exponential trend, while the stiffness profiles aligned with second-degree polynomials. Notably, both properties increased significantly within the first 6–7 µm from the surface.

**Figure 8:**
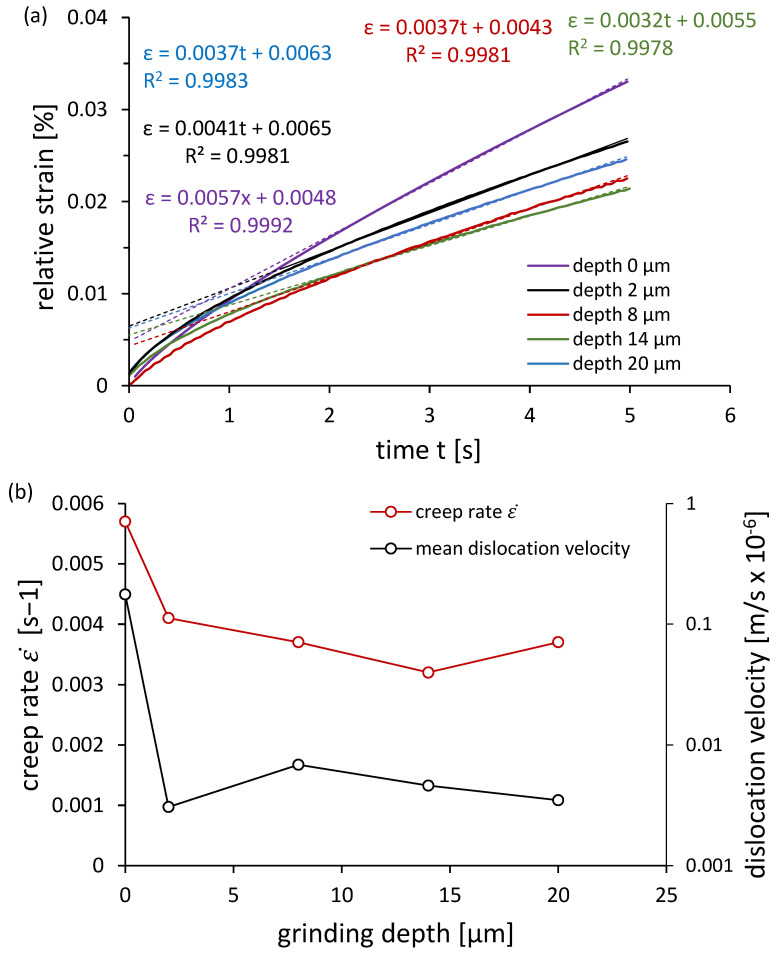
(**a**) Creep curves under a constant indenter load of 1366 mN and (**b**) changes in creep rate and average dislocation velocity as a function of grinding depth.

To analyze dislocation mobility, creep behavior under a constant load (1366 mN) was studied (Figure 8a). The steady-state creep rate was determined approximately 1.7 s after loading. The highest creep rate (0.0057 s^−1^) was observed in normalized steel, while the lowest (0.0032 s^−1^) occurred for the 14 µm depth. Using the Orowan equation (Equation (11)), the average dislocation velocity was calculated. Interestingly, despite higher dislocation density at 2 µm, the mean dislocation velocities for 2 µm and 20 µm depths were similar, suggesting an additional influence of residual stresses.

**Figure 9:**
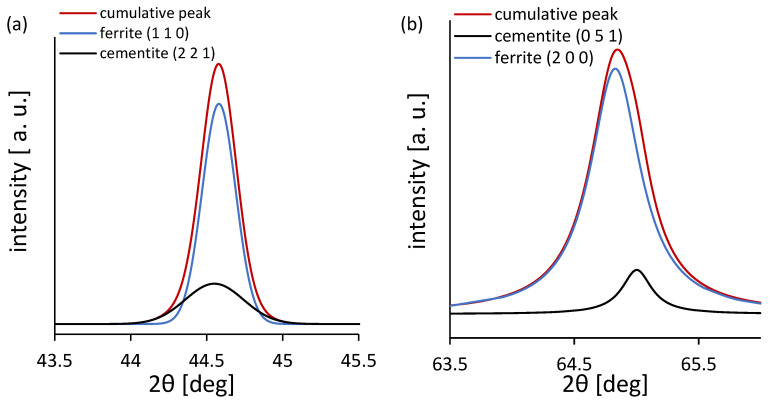

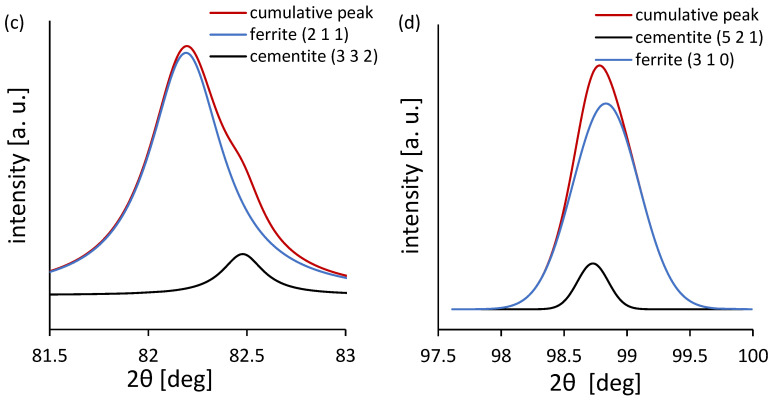
(**a**) Examples of deconvolution of overlapping peaks from ferrite and cementite for annealed C45 steel for angle 2θ about 44.5 degrees, (**b**) 65 degrees, (**c**) 82.3 degrees, and (**d**) 99 degrees.

**Figure 10:**
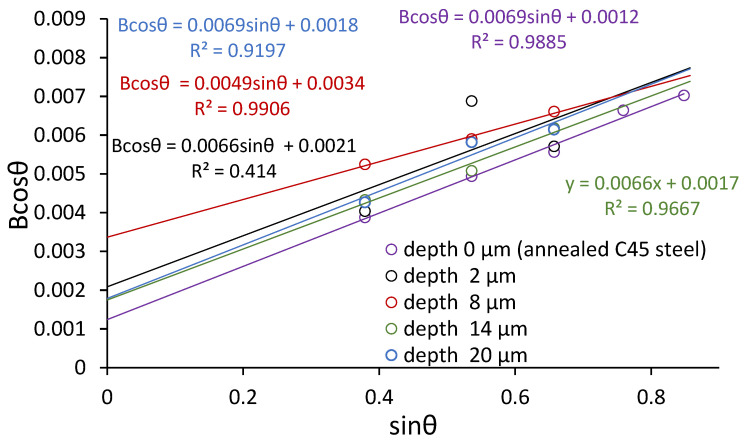
Plots of Bcosθ vs. sinθ for raw ferrite and after grinding on different depths.

To verify this, XRD analysis was performed using the Williamson–Hall method. Peak deconvolution was necessary due to an overlap of ferrite, cementite, magnetite, and hematite peaks (Figure 9). Figure 10 presents the dislocation density and crystallite size determined from XRD data using the Williamson–Hall method. The Bcosθ vs. sinθ plots for samples ground to depths of 2, 8, 14, and 20 µm are shown alongside the annealed (0 µm) C45 steel. Each data series was fitted with a linear regression, from which both the slope (related to the lattice microstrain) and intercept (inversely related to the crystallite size) were extracted. The highest slope was observed for the 2 µm depth, indicating the greatest microstrain and thus the highest dislocation density. This confirms that shallow grinding introduces significant lattice distortion. In contrast, the annealed steel shows the lowest strain values. The R^2^ values of the fits are generally high (except for the annealed state), indicating reliable correlation and fitting quality. These results confirm that grinding depth has a direct impact on the degree of crystal lattice distortion and microstructural refinement. It can be observed that the dislocation density reaches its maximum for the sample ground to a depth of 2 µm, which corresponds to the highest degree of plastic deformation and strain localization. As the grinding depth increases, the dislocation density decreases due to thermal recovery and reduced strain gradients. The crystallite size inversely correlates with dislocation density, confirming that refinement is driven by dislocation activity. These results are consistent with the findings of other researchers who observed similar dislocation behavior in ferritic steels subjected to surface plastic deformation [20,21,22]. The results obtained from the Williamson–Hall analysis are presented in Table 4.

**Figure 11:**
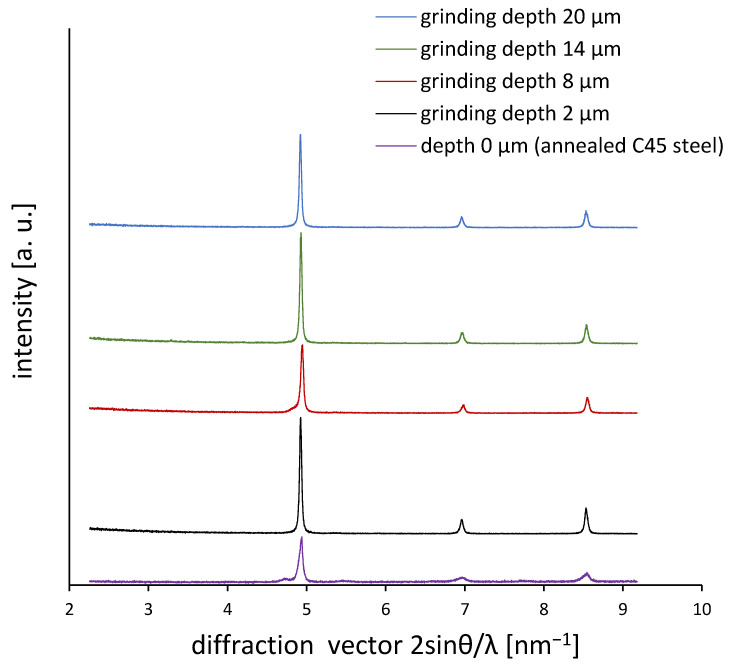
XRD diffractograms as a function of the diffraction vector K.

Figure 11 shows X-ray diffraction patterns (in reciprocal space, plotted as a function of diffraction vector 2sinθ/λ) for C45 steel samples ground to various depths (2, 8, 14, and 20 µm) as well as for the reference annealed sample. The sharp peaks observed in the annealed sample become noticeably broadened and less intense with a decreasing grinding depth, particularly for the 2 µm sample. This indicates significant lattice distortion, microstrain accumulation, and crystallite fragmentation caused by grinding-induced plastic deformation. These changes in the peak shape are consistent with the increased dislocation density inferred from Williamson–Hall analysis (Figure 10) and serve as a qualitative confirmation of microstructural refinement.

**Figure 12:**
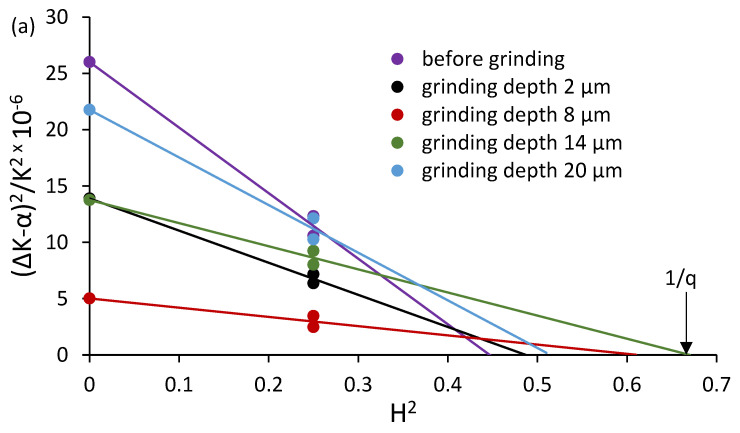

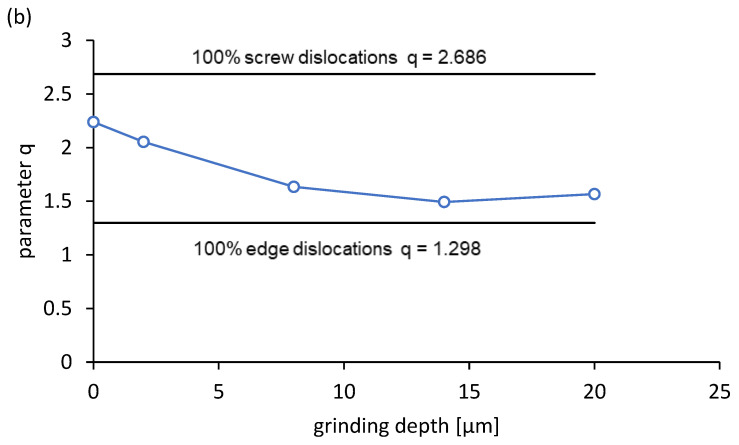
(**a**) The plot of Equation (6) for annealed ferrite and after grinding it to a different depth. The reciprocal of the intersect on the *H*^2^ axis gives 1/*q* and (**b**) the change in the *q* parameter value as a function of grinding depth.

Figure 12 presents the results of the analysis based on the empirical relationship proposed by Ungár, used to evaluate the character of dislocations in the surface layer after grinding. Part (a) shows plots of the (ΔK − α)^2^/K^2^ dependence as a function of H^2^ for annealed C45 steel and samples ground to various depths. The key parameter is the intersection point of the fitted lines with the H^2^ axis, which allows the determination of 1/q and thus the parameter q, indicating the relative contributions of screw and edge dislocations. The values of q are summarized in Figure 12b as a function of grinding depth. The highest q value (~2.1) was obtained for the sample ground to a depth of 2 µm, indicating a predominance of screw dislocations in the most severely plastically deformed surface layer. As the grinding depth increases, the q parameter gradually decreases, approaching an intermediate range between the theoretical values for pure screw (2.686) and edge (1.298) dislocations. This suggests that with an increasing grinding depth, the proportion of screw dislocations decreases, which can be attributed to the reduction in plastic strain gradients and partial reorganization of the dislocation structure in the deeper layers.

**Figure 13:**
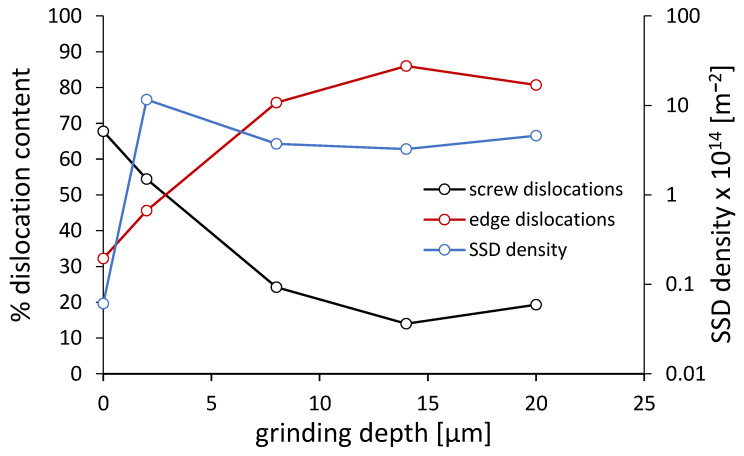
Changes in dislocation density and the percentage of screw and edge dislocations in ferrite after grinding to various depths.

Figure 13 complements the previous analysis by showing the evolution of statistically stored dislocation (SSD) density and the relative contribution of screw and edge dislocations in ferrite. The SSD density is plotted on the right axis (logarithmic scale), while the percentage of screw and edge dislocations is shown on the left axis. The highest SSD density is observed at a grinding depth of 2 µm, confirming intensive plastic deformation in the surface layer. At this depth, screw dislocations dominate, consistent with the high q value previously discussed. As the grinding depth increases, the proportion of screw dislocations decreases significantly, while edge dislocations become dominant at depths of 8–20 µm. This behavior is indicative of a shift in the prevailing slip systems and dislocation dynamics as the deformation becomes less localized and more thermally assisted. These findings further support the notion that the dislocation type and storage mechanisms are highly sensitive to grinding conditions, influencing the resulting hardening mechanisms in the surface layer.

## 4. Discussion

During grinding, the flow stress in the surface layer of steel is influenced by both the temperature and strain rate. In this study, it is assumed that the strain rate of ferrite remains constant across varying grinding depths. However, ferrite’s deformation behavior also depends on crystallographic orientation, as described by Schmid’s law [23]. Since microstructural evolution during grinding is governed by dislocation generation and annihilation, the resulting deformation behavior is directly related to the degree of deformation, i.e., the grinding depth. Accordingly, this section focuses exclusively on the influence of grinding depth.

In crystalline materials, plastic deformation typically occurs via dislocation glide along specific slip planes and directions. Although twinning is another deformation mechanism, it is rarely activated in BCC metals under ambient conditions, as it generally requires low temperatures or extremely high strain rates [24,25,26]. Dislocation climb, which is diffusion-controlled, becomes relevant only at elevated temperatures (>0.3 T_m_) or under high stresses [27,28], and it is therefore not considered here. Thus, dislocation glide is the dominant deformation mechanism during grinding [29].

In BCC metals such as ferrite, edge dislocations are first activated in slip systems with the lowest critical resolved shear stress. As deformation progresses, increasing dislocation density reduces mobility and impedes further glide. Continued deformation may activate systems with higher critical stresses. Ferrite has 48 slip systems: 12 {110}<111>, 12 {211}<111>, and 24 {321}<111>. Screw dislocations, by contrast, are not confined to specific planes and can glide in multiple directions.

Dislocations encounter both short-range and long-range barriers. Thermal activation enables them to overcome short-range obstacles, whereas long-range barriers require mechanical stress. Consequently, the total flow stress comprises a thermal component (σ*) and an athermal component (σ_i_), such that σ = σ* + σ_i_. The thermal component is described by the Orowan equation (Equation (11)), while the athermal component is governed by Taylor’s theory, which quantifies dislocation interactions (Equation (12)).

(12) $$\varepsilon_{i}=\alpha \cdot G\cdot b\sqrt{\rho_{SSD}}$$

Equations (11) and (12) indicate that increasing the dislocation density may either weaken the material (Orowan softening) or strengthen it (Taylor hardening). When dislocation mobility is high, softening prevails; when mobility is low, strengthening dominates. In BCC metals, deformation is highly temperature-sensitive and primarily limited by the low mobility of screw dislocations, which move significantly slower than edge dislocations [12,23,30].

At absolute zero, screw dislocations are straight. However, thermal fluctuations at elevated temperatures introduce kinks, preventing simultaneous motion along the entire dislocation line. Oppositely signed kinks create short mobile segments that propagate via diffusion, facilitating dislocation glide. The velocity of kink migration is expressed by the following:

(13) vk=DkεbhkT

where *D_k_* is the kink diffusion coefficient, *k* is Boltzmann’s constant, *b* Burgers vector, *h* the kink height, and *T* is temperature. The concentration of kinks *c_k_* depends on the periodicity of the dislocation line *d* and the kink formation energy Δ*F_k_* [31].

(14) ck=2dexp−ΔFkkT

Below the critical temperature *T_k_* (~350 K for α-Fe), dislocation motion occurs via kink-pair formation and propagation, significantly reducing mobility. Above *T_k_*, the yield strength becomes less temperature-dependent, and screw and edge dislocations exhibit similar mobilities [32,33]. Near free surfaces, screw dislocation mobility may increase due to kink source formation [30,34].

The present results confirm that the grinding depth exerts a pronounced influence on the dislocation structure, residual stress state, and mechanical response of C45 ferritic steel. While some trends agree with established models of surface hardening, others reveal atypical behaviors that require further interpretation.

At shallow grinding depths, where deformation is confined to a very thin surface layer, the strain rate is extremely high and thermal conductivity limits the temperature rise. These conditions are characteristic of grinding-induced severe plastic deformation. In BCC ferrite, such conditions promote the activation of screw dislocations over edge dislocations. Although edge dislocations have lower Peierls stress and are more mobile [12,23,30], screw dislocations dominate at high strain rates because their lower mobility becomes the rate-limiting factor in plastic deformation. The resulting dislocation pile-ups, together with high compressive residual stresses (~592 MPa), effectively restrict glide and promote forest hardening [35]. This microstructural state explains the observed maximum hardness and stiffness in the 2 µm layer, in agreement with findings from severe surface deformation techniques such as ultrasonic nanocrystal surface modification [36]. Moreover, the temperatures generated in the 2 µm ground layer did not exceed the critical threshold (T_k_ ≈ 350 K for α-Fe), above which screw dislocation mobility increases sharply due to kink-pair migration. Below this threshold, screw dislocations accumulate as kink propagation is thermally suppressed. This explains their predominance in the shallowest ground layers. The combined influence of high strain rates and subcritical temperatures thus directly favors the activation and accumulation of screw dislocations, which contribute to strengthening but also to reduced plasticity.

Moreover, the elevated compressive residual stresses observed at shallow and deep grinding depths played a dual role in the strengthening mechanism. First, they contributed to mobility restriction by promoting dislocation pile-ups and increasing the interaction stress, which hindered further glide. Second, they intensified strain gradients within the surface layer, thereby contributing to an increase in the density of geometrically necessary dislocations (GNDs). The combination of limited mobility and elevated GND density strongly correlates with the increase in hardness observed in nanoindentation results. These effects are particularly pronounced in the 2 µm ground layer, where the highest residual stress, dislocation density, and GND density were recorded, confirming the integral role of stress in strain hardening.

Although the present study focused primarily on dislocation-based mechanisms of surface hardening, it is important to note that the screw and edge dislocation fractions also have implications for other mechanical properties. Screw dislocations, due to their lower mobility and greater ability to cross-slip, can act as obstacles to crack propagation, potentially contributing to enhanced fracture toughness and fatigue resistance. Edge dislocations, which are more mobile, tend to dominate uniform plastic deformation and influence the strain hardening capacity. The observed predominance of screw dislocations in shallowly ground layers may therefore not only contribute to hardness but also suggest improved resistance to cyclic loading or crack growth, an aspect that could be explored in future mechanical testing.

With increasing grinding depth to 8–14 µm, hardness and residual stress decrease, consistent with partial thermal recovery. The higher grinding energy at these depths elevates surface temperature, facilitating dislocation rearrangement and, in some cases, the formation of continuous oxide films. These thermal effects, coupled with a shift toward a higher fraction of mobile edge dislocations, reduce overall strengthening. Such behavior is consistent with earlier reports that prolonged or deeper grinding promotes recovery and reduces strain gradients in ferritic steels [37].

An exception to the expected trend occurs at 20 µm, where hardness is higher than at intermediate depths despite significant thermal input and a predominance of edge dislocations. This anomaly can be partly explained by the simultaneous presence of very high compressive residual stresses (>600 MPa) and stresses arising from the oxide–metal interface, particularly where hematite is present. Hematite’s rhombohedral structure imposes greater lattice mismatch with ferrite than magnetite, which can locally pin dislocations and hinder glide, producing a stress-assisted hardening effect similar to that reported in steels subjected to high-intensity shot peening at elevated surface temperatures [38,39]. However, although the 20 µm layer exhibits high residual stress, its hardness remains lower than that of the 2 µm sample. The substantial temperature rise at greater grinding depths, evidenced by the presence of both magnetite and hematite and indicating surface temperatures above 623 K, likely promotes partial dynamic recovery and even recrystallization, reducing dislocation density and annihilating substructures formed during deformation. Moreover, the predominance of more mobile edge dislocations under elevated thermal conditions makes them less effective at impeding plastic flow compared to immobile screw dislocations. As a result, the strain and stress-induced hardening in the 20 µm layer are partially offset by thermally induced softening, explaining why the 2 µm sample, subjected to a lower temperature and dominated by screw dislocations, achieves higher hardness despite lower thermal exposure.

To assess the long-term applicability of the grinding-induced surface structure, it is also important to consider its stability during service. Over extended operational periods, particularly under cyclic loading or elevated temperatures, partial stress relaxation and dislocation rearrangement may occur. This could gradually reduce surface hardness and affect the balance between screw and edge dislocation populations. However, for moderate service temperatures below 400 K, the likelihood of significant recovery or recrystallization remains low, suggesting that the strain-hardened structure produced by shallow grinding will retain its beneficial properties over time. Further studies involving aging, creep, or fatigue exposure would help validate this long-term stability and are recommended for future work.

The observed evolution of the dislocation character with the grinding depth reflects the interplay between the strain rate, temperature, and deformation geometry. Shallow grinding confines deformation to a thin layer, producing steep strain gradients and enhancing the formation of geometrically necessary dislocations (GNDs), under which conditions screw dislocations dominate, limiting plasticity but maximizing strengthening. In contrast, deeper grinding distributes deformation over a larger volume, increases the contribution of statistically stored dislocations (SSDs), and promotes edge dislocation activity, which supports more uniform plastic flow. This transition in the dislocation character aligns with the lever rule analysis of the q parameter. The combined EBSD and XRD analyses confirm that the scale of microstructural refinement also depends on the grinding depth: EBSD revealed increased grain orientation spread (GOS) and misorientation of grain boundaries (MOS) in shallow layers, indicative of subgrain formation and dislocation cell structures, while XRD-derived crystallite sizes (45–128 nm) reveal nanoscale coherent domains, even though they do not directly correspond to EBSD-measured grain sizes. Such refinement mechanisms are well-recognized contributors to strengthening in ferritic steels processed by surface mechanical treatments [26,34]. The nanoindentation results (Figure 6, Table 1) further show that the increase in hardness near the surface correlates with both GND and SSD densities: GNDs, arising from steep strain gradients, are particularly pronounced in shallowly ground layers, with the highest density at 2 µm, while SSDs, representing the cumulative dislocation content from uniform plastic deformation, also peak at this depth. The synergy of GND and SSD accumulation, combined with substructural refinement (increased GOS and MOS), provides a comprehensive explanation for the substantial surface hardening effect in the shallowest ground layers. It should also be noted that the crystallite sizes obtained from the Williamson–Hall method (ranging from ~45 to 128 nm) represent the size of coherently diffracting domains and are therefore significantly smaller than the grain sizes identified in EBSD measurements. While EBSD captures microstructural grains in the micrometer range, the reduced domain sizes revealed by XRD are consistent with the formation of subgrain structures and dislocation cells. Although a direct numerical comparison is not possible due to the differing resolution and sensitivity of the techniques, the EBSD results qualitatively confirm the grain fragmentation and increased misorientation suggested by the XRD-based analysis, supporting the reliability of the observed microstructural refinement.

Overall, the present findings show that shallow grinding maximizes surface strengthening through a synergy of screw dislocation accumulation, high GND density, and compressive residual stress buildup, while avoiding excessive thermal recovery. At intermediate depths, the thermal effects reduce strengthening, whereas at the deepest tested depth (20 µm), residual and oxide-induced stresses partially compensate for recovery losses, producing a secondary hardness peak. This complex interplay of mechanical and thermal mechanisms underscores the importance of precise grinding depth control when targeting specific surface property profiles. From a broader perspective, these results extend the current understanding of grinding-induced hardening in ferritic steels by quantitatively linking the dislocation character, residual stress, and microstructural refinement to the grinding depth. The anomaly observed at 20 µm highlights the need for further work to isolate the role of oxide-phase-induced stresses and to assess the stability of such hardening under cyclic loading or elevated-temperature service, with potential implications for the development of optimized, environmentally friendly grinding parameters as an alternative to thermochemical hardening. In summary, shallow grinding promotes ferrite strengthening via multiple mechanisms: forest hardening [40], GND and SSD accumulation, and substructural refinement. While thermal activation may enhance dislocation mobility in deeper ground layers, the dominant hardening in shallow regions is attributed to dislocation interactions and residual stress buildup. Since grinding temperatures remained below the critical transformation thresholds, softening from recovery or recrystallization was avoided, enabling preservation of the strain-hardened surface structure [41,42].

## 5. Conclusions

This study demonstrates that shallow grinding at a depth of 2 μm significantly enhances the hardness of C45 steel due to the combined effects of microstructural refinement and dislocation-based mechanisms. The main conclusions are as follows:

Shallow grinding depths of ~2 µm provide the highest surface hardness and strengthening effect in C45 ferritic steel, resulting from the combined action of immobile screw dislocations, high GND density, and significant compressive residual stresses. This condition is optimal when the aim is to maximize wear resistance and surface load-bearing capacity without altering bulk properties.

1. Intermediate grinding depths of 8–14 µm reduce hardness due to partial thermal recovery and increased edge dislocation mobility. These parameters are recommended when a balance between surface strengthening and ductility is desired, for example in components subjected to both wear and impact loads.
2. Deep grinding (20 µm) produces a secondary hardness peak due to high residual and oxide-induced stresses, but the stability of this effect is lower because of thermally activated recovery. Such conditions may be suitable for short-term performance enhancement but require caution for long-term applications, especially at elevated temperatures or under cyclic loading.
3. The results show that shallow grinding (2 µm) produces the highest hardness, highest GND and SSD densities, and the most stable compressive residual stresses. Such a microstructural state is expected to improve resistance to surface-initiated fatigue, making this depth preferable in applications where both high hardness and structural stability are critical.
4. The observed reduction in hardness at intermediate and deep grinding depths is associated with thermally activated recovery, as confirmed by oxide phase composition and dislocation character. These findings indicate that maintaining grinding temperatures below the oxide formation threshold corresponding to hematite appearance (~623 K) helps preserve the strain-hardened microstructure.
5. The experimentally established relationships between the grinding depth, dislocation type, residual stress magnitude, and microstructural refinement provide a quantitative basis for selecting grinding parameters to achieve targeted hardness profiles. This approach offers a controllable, mechanical alternative to thermochemical treatments for enhancing surface performance of ferritic steels.

## Figures and Tables

**Table 1:** Chemical composition of C45 steel.

Chemical Composition wt.%							
C	Mn	Si	P	S	Cu	Cr	Ni
0.47	0.65	0.27	0.030	0.025	0.25	0.17	0.26

**Table 2:** Grinding parameters used in the study.

Parameter	Symbol	Value	Unit	Remarks
Grinding machine	–	SPG 25 × 60	–	CNC surface grinder
Grinding wheel	–	Norton 38A60LVS	–	Vortex type
Wheel dimensions	–	250 × 25 × 76.2	mm	Outer × Width × Bore
Circumferential speed	v_s_	23	m/s	Constant
Table feed rate	v_ft_	1	m/min	Constant
Number of passes	–	4	–	Two per each side
Grinding depth per pass	a_e_	2/8/14/20	µm	Variable
Coolant	–	Water-based emulsion	–	Used during grinding
Wheel dressing	–	Before each trial	–	To maintain wheel sharpness

**Table 3:** Values characterizing surface layers after grinding to different depths, obtained in the nanoindentation test.

	*h_c_*	*ρ_GND_*	*H_ISE_*	*ρ_SSD_*	*H_0_*	E*	$\dot{\varepsilon }$	*υ*
	(nm)	(m^−2^) × 10^14^	(GPa)	(m^−2^) × 10^15^	(GPa)	(GPa)	(s^−1^)	(nm/s)
depth 0 µm	754.12	0.84	1.44 ± 0.13	0.114	1.09	87.9 ± 7.9	0.0057	176.06
depth 2 µm	337.64	1.88	7.16 ± 0.82	4.716	7.02	334.0 ± 56.8	0.0041	3.061
depth 8 µm	419.97	1.51	4.63 ± 0.59	1.899	4.46	235.9 ± 30.1	0.0037	6.861
depth 14 µm	395.44	1.61	5.22 ± 0.77	2.446	5.06	255.0 ± 33.8	0.0032	4.607
depth 20 µm	357.12	1.78	6.40 ± 0.49	3.740	6.25	345.7 ± 45.1	0.0037	3.483

**Table 4:** Microstrain, crystallite size, and residual stress in raw ferrite and after grinding to different depth.

	4ε (%)	a_s_·λ/L	L (nm)	E (GPa)	σ_R_ (MPa)
annealed ferrite	0.0069	0.0012	128.3333	87.9	151.63
2 µm depth	0.0066	0.0021	73.33333	358.99	592.33
8 µm depth	0.0049	0.0034	45.29412	215.48	263.96
14 µm depth	0.0066	0.0017	90.58824	237.70	392.21
20 µm depth	0.0069	0.0018	85.55556	350.85	605.22

## Data Availability

The research data are available from the corresponding author upon reasonable request.

## References

[1] Hahn R. (1981). The Influence of Threshold Forces on Size Roundness and Contour Errors in Precision Grinding. CIRP Ann..

[2] Rowe W.B. (2009). Grinding Machine Developments. Principles of Modern Grinding Technology.

[3] Heinzel C., Bleil N. (2007). The Use of the Size Effect in Grinding for Work-hardening. CIRP Ann..

[4] Brinksmeier E., Giwerzew A. (1999). Theoretical Estimation of the Maximum Contact Area Temperature in Shave-Grinding. Prod. Eng..

[5] Bardin J.A., Eisen E.A., Tolbert P.E., Hallock M.F., Hammond S.K., Woskie S.R. (1997). Mortality Studies of Machining Fluid Exposure in the Automobile Industry V: A Case-Control Study of Pancreatic Cancer. Am. J. Ind. Med..

[6] Szkodo M., Chodnicka-Wszelak K., Deja M., Stanisławska A., Bartmański M. (2020). The Influence of the Depth of Cut in Single-Pass Grinding on the Microstructure and Properties of the C45 Steel Surface Layer. Materials.

[7] Malkin S., Guo C. (2008). Grinding Technology: Theory and Application of Machining with Abrasives.

[8] Williamson G.K., Hall W.H. (1953). X-ray Line Broadening from Filed Aluminium and Wolfram. Acta Metall..

[9] Révész Á., Ungár T., Borbély A., Lendvai J. (1996). Dislocations and Grain Size in Ball-Milled Iron Powder. Nanostruct. Mater..

[10] Cong Z., Murata Y. (2011). Dislocation Density of Lath Martensite in 10Cr-5W Heat-Resistant Steels. Mater. Trans..

[11] HajyAkbary F., Sietsma J., Böttger A.J., Santofimia M.J. (2015). An Improved X-ray Diffraction Analysis Method to Characterize Dislocation Density in Lath Martensitic Structures. Mater. Sci. Eng. A.

[12] Lim H., Hale L.M., Zimmerman J.A., Battaile C.C., Weinberger C.R. (2015). A Multi-Scale Model of Dislocation Plasticity in α-Fe: Incorporating Temperature Strain Rate and Non-Schmid Effects. Int. J. Plast..

[13] Johnson K.L. (1985). Contact Mechanics.

[14] Arsenlis A., Parks D.M. (1999). Crystallographic Aspects of Geometrically-Necessary and Statistically-Stored Dislocation Density. Acta Mater..

[15] Qiu X., Huang Y., Nix W.D., Hwang K.C., Gao H. (2001). Effect of Intrinsic Lattice Resistance in Strain Gradient Plasticity. Acta Mater..

[16] Durst K., Backes B., Franke O., Göken M. (2006). Indentation Size Effect in Metallic Materials: Modeling Strength from Pop-In to Macroscopic Hardness Using Geometrically Necessary Dislocations. Acta Mater..

[17] Taylor G.I. (1938). Plastic Strain in Metals. J. Inst. Met..

[18] Orowan E. (1934). Zur Kristallplastizität. III. Z. Phys..

[19] Dyson B.F. (2009). Microstructure-Based Creep Constitutive Model for Precipitation Strengthened Alloys: Theory and Application. Mater. Sci. Technol..

[20] Ghara T., Paul S., Bandyopadhyay P.P. (2021). Influence of Grit Blasting on Residual Stress Depth Profile and Dislocation Density in Different Metallic Substrates. Metall. Mater. Trans. A.

[21] Torabian N., Favier V., Dirrenberger J., Adamski F., Ziaei-Rad S., Ranc N. (2017). Correlation of the high and very high cycle fatigue response of ferrite based steels with strain rate-temperature conditions. Acta Mater..

[22] Abed F.H., Saffarini M.H., Abdul-Latif A., Voyiadjis G.Z. (2017). Flow Stress and Damage Behavior of C45 Steel Over a Range of Temperatures and Loading Rates. J. Eng. Mater. Technol..

[23] Po G., Cui Y., Rivera D., Cereceda D., Swinburne T.D., Marian J. (2016). A Phenomenological Dislocation Mobility Law for BCC Metals. Acta Mater..

[24] Wang J., Zeng Z., Weinberger C.R., Zhang Z., Zhu T., Mao S.X. (2015). In Situ Atomic-Scale Observation of Twinning-Dominated Deformation in Nanoscale Body-Centred Cubic Tungsten. Nat. Mater..

[25] Li X., Zhang Z., Wang J. (2023). Deformation Twinning in Body-Centered Cubic Metals and Alloys. Prog. Mater. Sci..

[26] Gao Y., Zhang Y., Wang Y. (2020). Determination of Twinning Path from Broken Symmetry: A Revisit to Deformation Twinning in BCC Metals. Acta Mater..

[27] Carrez P., Mussi A., Cordier P. (2024). On Dislocation Climb as an Important Deformation Mechanism for Planetary Interiors. Annu. Rev. Earth Planet. Sci..

[28] Huang M., Li Z., Tong J. (2014). The Influence of Dislocation Climb on the Mechanical Behavior of Polycrystals and Grain Size Effect at Elevated Temperature. Int. J. Plast..

[29] Zhou X., Wang X., Fey L., He S., Beyerlein I., Cao P., Marian J.O. (2023). Models of Dislocation Glide and Strengthening Mechanisms in BCC Complex Concentrated Alloys. MRS Bull..

[30] Kaufmann D., Schneider A.S., Mönig R., Volkert C.A., Kraft O. (2013). Effect of Surface Orientation on the Plasticity of Small BCC Metals. Int. J. Plast..

[31] Vitek V. (2004). Core Structure of Screw Dislocations in Body-Centred Cubic Metals: Relation to Symmetry and Interatomic Bonding. Philos. Mag..

[32] Seeger A., Holzwarth U. (2006). Slip Planes and Kink Properties of Screw Dislocations in High-Purity Niobium. Philos. Mag..

[33] Greer J.R., Weinberger C.R., Cai W. (2008). Comparing the Strength of F.C.C. and B.C.C. Sub-Micrometer Pillars: Compression Experiments and Dislocation Dynamics Simulations. Mater. Sci. Eng. A.

[34] Matsui H., Kimura H. (1973). A Mechanism of the “Unexpected {110} Slip” Observed in BCC Metals Deformed at Low Temperatures. Scr. Metall..

[35] Kapci M.F., Schön J.C., Bal B. (2021). The Role of Hydrogen in the Edge Dislocation Mobility and Grain Boundary–Dislocation Interaction in α-Fe. Int. J. Hydrogen Energy.

[36] Lu K., Lu J. (2004). Nanostructured surface layer on metallic materials induced by surface mechanical attrition treatment. Mater. Sci. Eng. A.

[37] Wen J., Tang J., Shao W., Zhou W., Huang W. (2023). Towards Understanding Subsurface Characteristics in Burn Process of Gear Profile Grinding. Materials.

[38] Gaviria J., Bohé A., Pasquevich A., Pasquevich D. (2007). Hematite to Magnetite Reduction Monitored by Mössbauer Spectroscopy and X-ray Diffraction. Phys. B Condens. Matter.

[39] Yaghoobi M., Voyiadjis G.Z. (2018). The Effects of Temperature and Strain Rate in FCC and BCC Metals During Extreme Deformation Rates. Acta Mater..

[40] Yao S., Pei X., Yu J., Yu Y., Wu Q. (2018). Homogeneous Nucleation and Forest Hardening Result in Thermal Hardening Phenomenon in Shock Loaded BCC Metals. arXiv.

[41] Grabowski B., Zotov N. (2021). Thermally-Activated Dislocation Mobility in BCC Metals: An Accelerated Molecular Dynamics Study. Comput. Mater. Sci..

[42] Eleti R.R., Stepanov N., Zherebtsov S. (2020). Mechanical Behavior and Thermal Activation Analysis of HfNbTaTiZr Body-Centered Cubic High-Entropy Alloy During Tensile Deformation at 77 K. Scr. Mater..

